# Melanoma Identification and Management in an Unsheltered Male Using Teledermatology: Street Medicine Perspective

**DOI:** 10.2196/42113

**Published:** 2022-11-04

**Authors:** Emily Eachus, Taha Rasul, Armen Henderson

**Affiliations:** 1 University of Miami Miller School of Medicine Miami, FL United States; 2 Jackson Memorial Hospital Miami, FL United States

**Keywords:** skin cancer, REDCap, homelessness, melanoma, teledermatology, street medicine, dermatology, homeless, case report, case study, skin lesion, biopsy, dermatologist, insurance, low income, health coverage, skin, cancer

## Abstract

Skin cancers are concerning for unsheltered people experiencing homelessness because of their high levels of sun exposure. Currently, there is little data on the prevalence of skin cancers in people experiencing homelessness. Skin diseases are often untreated in people experiencing homelessness due to a lack of access to specialized care. Miami Street Medicine (MSM) is an organization that provides people experiencing homelessness in the Miami Health District with medical care in a nonclinical street setting, near overpasses, sidewalks, and encampments. We present a case of an unsheltered 59-year-old male with a pigmented, 2 cm × 2 cm facial lesion that developed over several years. Through a teledermatology consultation, his lesion was highly suspicious of melanoma and further evaluation was recommended. Due to a lack of insurance, he could not be treated at any dermatology clinic. Coincidentally, 2 weeks later, he developed cellulitis of his lower extremity and was admitted to the local safety-net hospital through the emergency department. By coordinating with his primary inpatient team, MSM was able to include a biopsy of the lesion as part of his hospital stay. The results demonstrated melanoma in situ. The vital course of action was to ensure treatment before metastasis. After registration for insurance and follow-up with a surgical oncology team, he is weeks away from excision and reconstruction surgery. His unsheltered status made follow-up difficult, but MSM bridged the gap from the street to the clinical setting by incorporating teledermatology into patient evaluations and leveraging connections with community shareholders such as charitable clinics and volunteer physicians. This case also represents the barriers to care for cancer-based dermatologic outreach among people experiencing homelessness.

## Introduction

People experiencing homelessness are a high-risk patient population with suboptimal health outcomes. People experiencing homelessness can be sheltered, meaning they are living in a temporary housing facility like a homeless shelter, or unsheltered, meaning they do not have any temporary housing and are living outside. By some estimates, the average life expectancy of people experiencing homelessness is 55 years, more than 20 years below the United States national average [1]. People experiencing homelessness are more likely to have decreased access to longitudinal care while also having a high burden of HIV, respiratory illnesses, chronic liver diseases, and severe skin diseases [2,3]. In 2019, there were an estimated 580,000 sheltered and unsheltered people experiencing homelessness in the United States, which has likely increased due to the SARS-CoV-2 pandemic [4,5]. The resulting fallout from the loss of employment and evictions has only served to worsen the homelessness crisis. There are fewer social services offered to people experiencing homelessness due to concerns over close contact, and programs geared specifically toward medical outreach in people experiencing homelessness communities continue to suffer from scarce funding [5,6]. As a result, people experiencing homelessness lack access to basic primary care and specialized dermatologic care.

Unsheltered people experiencing homelessness in regions like the Southern United States have high levels of sun exposure, which increases their risk of developing skin cancer. However, there is still insufficient data on skin health and cancer parameters in this vulnerable population.

People experiencing homelessness with lesions suspicious of skin cancers are often unable to receive timely evaluation due to a lack of insurance, transportation, or funds. Commonly comorbid health conditions such as substance use disorders, soft tissue infections, lung diseases, and mental health issues may also lead to primary physicians prioritizing those over skin cancer screening [7].

Street medicine is a model of direct care where providers see people experiencing homelessness at encampments, sidewalks, and overpasses. This is different from the common free clinic model because street medicine brings providers directly to the patients rather than vice versa [7]. These patients live in abject poverty, lacking food, shelter, and medication, as well as the means to coordinate formal health care visits. It has often been called “house calls for the homeless” and represents a shift in the delivery of health care to the most socially and medically vulnerable patients.

This case describes encountering a 59-year-old unsheltered male with a suspicious pigmented lesion that was later found to be melanoma and the notable barriers to care that prevented timely evaluation. This also highlights how the street outreach model used by Miami Street Medicine (MSM), in conjunction with teledermatologist consultation, connected this patient to specialized care for the comprehensive evaluation of his melanoma.

## Case Report

**Figure 1:**
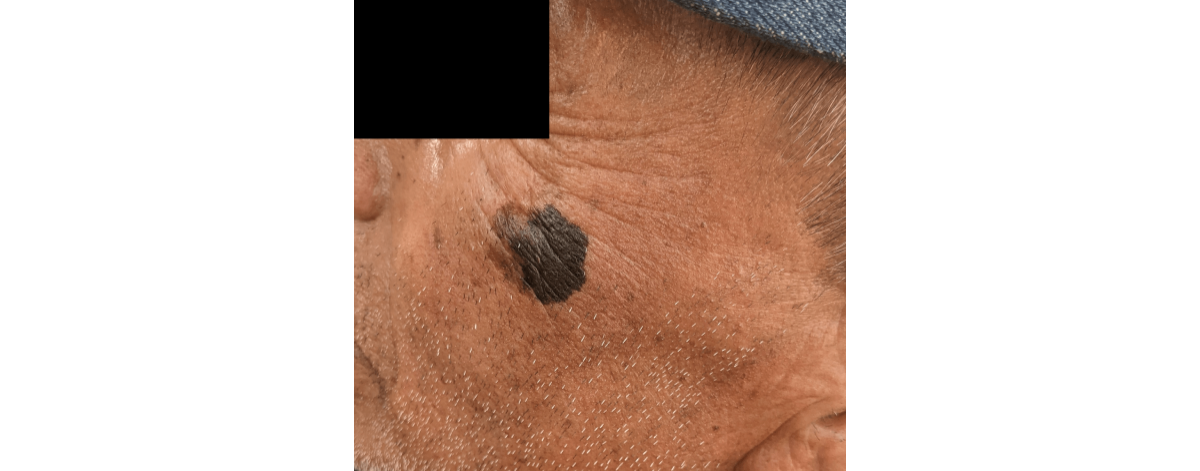
Pigmented 2 cm × 2 cm lesion noted, later biopsied and confirmed to be melanoma in situ.

During regular street outreach, a 59-year-old Spanish-speaking man with a history of hypertension, poorly managed type 2 diabetes mellitus, and venous stasis was encountered. He was wheelchair-bound as a result of being hit by a car a year prior and displayed limited reading literacy in English or Spanish. After taking a guided history focused on preventable health problems, he was noted to have a dark brown, irregular, 2 cm × 2 cm patch on his left upper cheek (Figure 1).

He was unsure of when this lesion started growing and did not endorse any itching or bleeding. Three years prior, he had been previously seen by a charity-based primary provider without any appropriate workup. The suspicious character of the lesion warranted documentation and a tele-consult with a dermatologist. The tele-consult was done using a modified secure medical data collecting application called REDCap. Through this software, physicians could assess photos of the lesion and consider the need for a biopsy.

**Figure 2:**
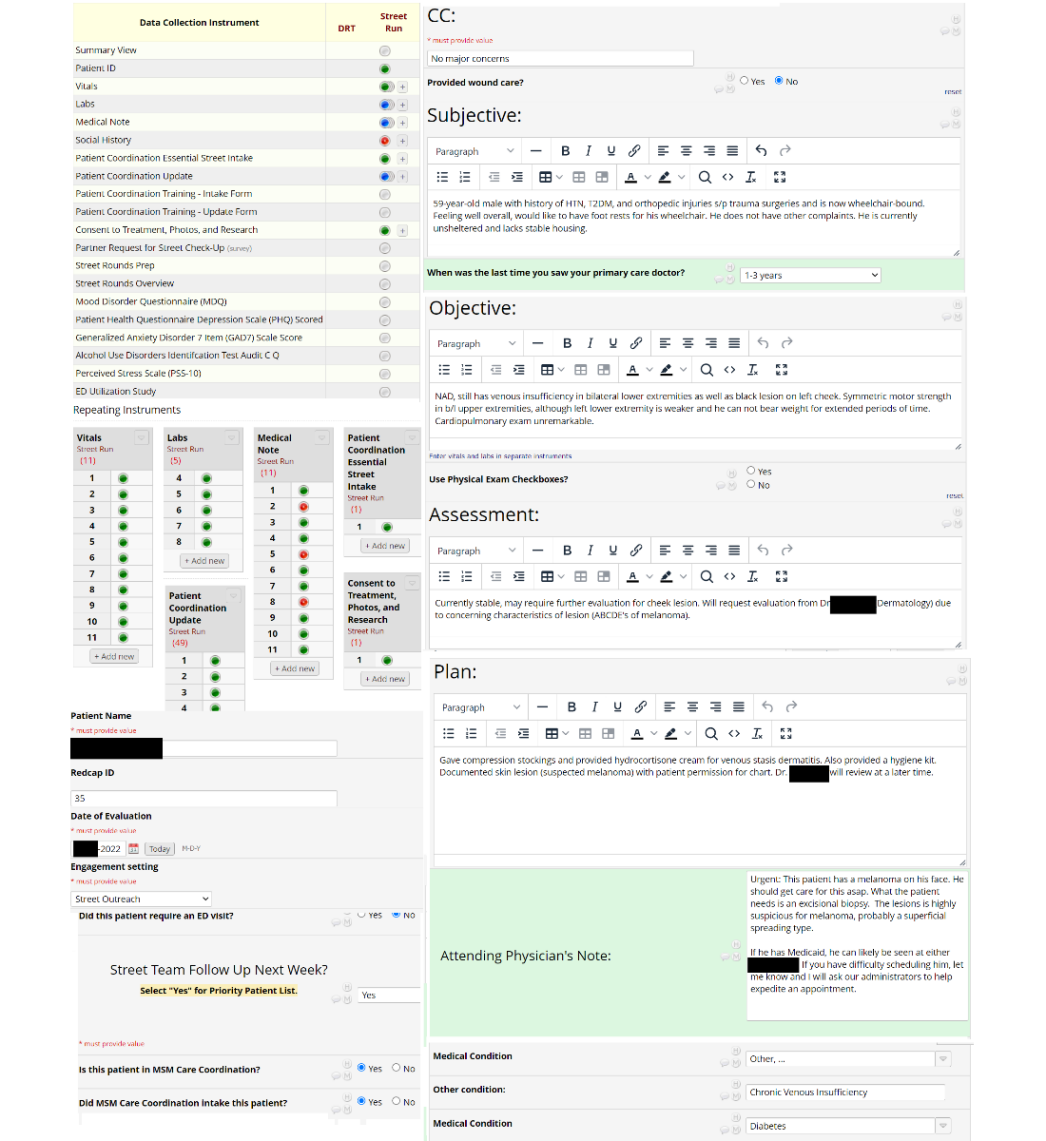
The layout of the REDCap-based medical record for this patient’s initial evaluation. There are multiple documented instances of vitals, labs, photographs, and medical notes from providers, reflecting a continued relationship with the patient. The Plan section included a dermatology evaluation, which was then completed according to the Attending Physician’s Note. HTN: hypertension; MSM: Miami Street Medicine; T2DM: type 2 diabetes mellitus.

A custom electronic medical record for street medicine use was developed in REDCap. This stored patient data in a confidential manner for team members to review patient charts and coordinate with other providers (Figure 2). Special emphasis is given to social history as it can provide a more complete picture of the patient’s housing status and comorbidities like alcohol use disorder. After inputting the data into our custom REDCap, an attending dermatologist was requested to view the chart containing the medical history and images of the patient’s lesion.

The team was informed that this lesion had a high probability of being melanoma and to seek further evaluation promptly. However, his lack of insurance made an outpatient dermatology clinic visit impossible. The street medicine team continued to maintain a close level of follow-up while also working toward some form of Medicaid enrollment. Our patient had reliable access to a phone and communicated with the team regularly about where to meet next and any questions after the first visit.

Unfortunately, 2 weeks after the initial evaluation, the patient presented to the emergency department with a soft tissue infection of the lower extremity. The severity of his condition necessitated hospitalization and intravenous antibiotic administration.

**Figure 3:**
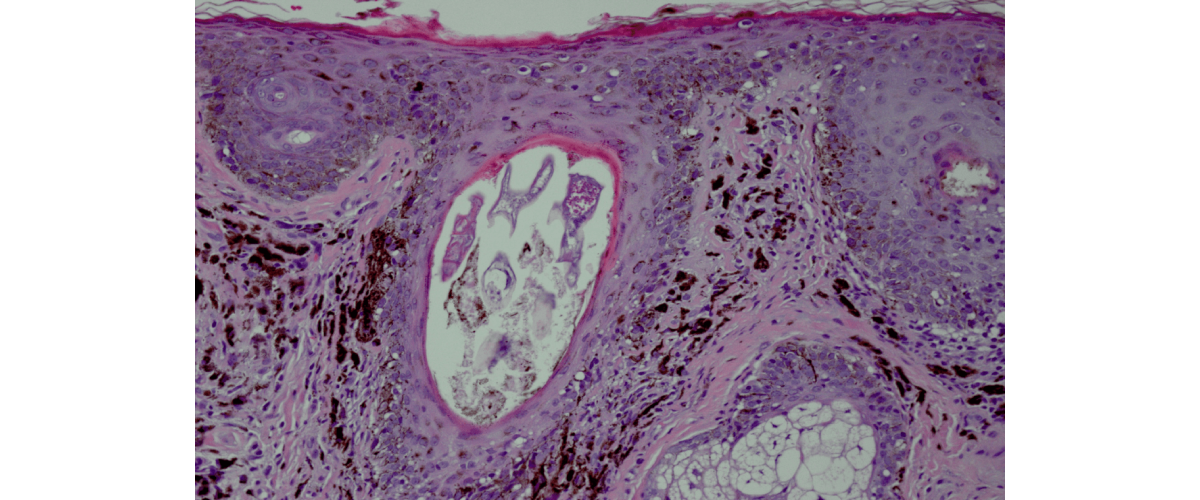
Pathology slide of pigmented lesion. Numerous dermal macrophages with an increased number of melanocytes along the dermal-epidermal junction and extending down hair follicles. Melanoma in situ extending to peripheral edges.

MSM providers established contact with the patient and later his primary inpatient team to arrange for a formal dermatological consult and skin biopsy. Two punch biopsies were obtained from the lesion, confirming a diagnosis of melanoma in situ (Figure 3).

Additionally, the MSM team expedited his Medicaid enrollment to cover his much-needed treatment. Given the size and sensitive location of the lesion, our team also coordinated with surgical oncology and plastic surgery providers on his behalf for management at a later date. After discharge, our patient was at risk of being lost to follow-up due to his unstable housing situation, lack of funds, and limited health literacy. Transportation was arranged so that he could make his appointments, and street medicine providers followed up regularly in a street setting and via telephone calls to assess his overall status. He attended his first set of appointments, which involved circumferential biopsy for staging through the staged marginal and central excision method [8]. He is currently being cleared for surgery, which will involve excision and reconstruction.

He also continues to be followed by the street medicine team to ensure that he can follow up appropriately, as well as for the management of his other health issues.

## Discussion

MSM participated in the care of the patient before, during, and after hospitalization. Timely intervention by MSM led to the diagnosis and ongoing management of melanoma-in-situ in a patient experiencing homelessness. Many people experiencing homelessness do not have access to consistent medical care, which can delay the diagnosis of their illnesses [9]. Care is often unaffordable and navigating the health care system while being homeless without reliable access to technology (eg, phones and the internet) is extremely difficult. In this case, he was evaluated by dermatology providers due to being admitted for an unrelated condition. The patient had access to a phone, which greatly streamlined care coordination for treatment. Unfortunately, many people experiencing homelessness do not have reliable access to phones or other technology, which makes further coordination challenging. Our only source of communication with phoneless patients is through weekly street encounters.

Our patient’s story also highlights a common scenario for people experiencing homelessness: inpatient hospitalization often being the only way to access specialty care.

However, teledermatology evaluation through REDCap provided a valuable consultation that guided further treatment. Our custom REDCap database provides a secure yet accessible medical record for patient care. The evaluation completed through teledermatology in this setting allowed a systematic relay of information from the consultant dermatologist to the rest of the care team. This can be a valuable adjunct to standard street medicine projects as it provides a customizable framework depending on patient needs, especially for resource-limited settings [10].

The unfortunate reality of our patient’s story is that it may be one of many underdocumented instances of vulnerable patients being lost to follow-up in the current system. In our patient’s case, issues with insurance, funding, transportation, and even understanding of discharge instructions meant that, had the MSM team not followed him longitudinally, his cancer would have remained untreated. The imperfect system of health care and human rehabilitation leaves notable barriers that may not be resolved until there is a fundamental inclusion of the health of society’s most vulnerable [11]. Until then, organizations like Miami Street Medicine have no choice but to step in and try to bridge glaring defects in care for the homeless.

### Conclusions

This 59-year-old unsheltered patient with multiple comorbidities was successfully screened and evaluated for his melanoma before it metastasized. Instead of the traditional free clinic model where patients come to the provider, initiatives like street medicine can directly provide screening and care for unsheltered patients who are unable to attend such clinics. Even so, the street medicine team had to leverage connections with the medical community to coordinate care. The technology used by MSM, such as REDCap, provided another way to connect people experiencing homelessness to care via tele-consults. In the current health care model where people experiencing homelessness face difficulties in longitudinal care, street-based outreach can be a valuable tool for establishing a sustained connection, thereby improving follow-up.

## Abbreviations

MSM: Miami Street Medicine
